# Mutation in a SARS-CoV-2 Haplotype from Sub-Antarctic Chile Reveals New Insights into the Spike’s Dynamics

**DOI:** 10.3390/v13050883

**Published:** 2021-05-11

**Authors:** Jorge González-Puelma, Jacqueline Aldridge, Marco Montes de Oca, Mónica Pinto, Roberto Uribe-Paredes, José Fernández-Goycoolea, Diego Alvarez-Saravia, Hermy Álvarez, Gonzalo Encina, Thomas Weitzel, Rodrigo Muñoz, Álvaro Olivera-Nappa, Sergio Pantano, Marcelo A. Navarrete

**Affiliations:** 1 Escuela de Medicina, Universidad de Magallanes, Punta Arenas 6210427, Chile; jorge.gonzalez@umag.cl (J.G.-P.); monica.pinto@umag.cl (M.P.); diego.alvarez@umag.cl (D.A.-S.); hermy.alvarez@umag.cl (H.Á.); rodrigo.munoz@umag.cl (R.M.); 2 Centro Asistencial Docente y de Investigación, Universidad de Magallanes, Punta Arenas 6210005, Chile; 3 Departamento de Ingeniería en Computación, Universidad de Magallanes, Punta Arenas 6210427, Chile; jacqueline.aldridge@umag.cl (J.A.); marco.montes@umag.cl (M.M.d.O.); roberto.uribe@umag.cl (R.U.-P.); 4 Enfermedades Infecciosas, Hospital Clínico de Magallanes, Punta Arenas 6210005, Chile; 5 Departamento de Matemática y Física, Universidad de Magallanes, Punta Arenas 6210427, Chile; jose.fernandezg@umag.cl; 6 Centro de Genética y Genómica, Instituto de Ciencias e Innovación en Medicina, Facultad de Medicina Clínica Alemana, Universidad del Desarrollo, Santiago 7610658, Chile; gencina@udd.cl; 7 Laboratorio Clínico, Clínica Alemana de Santiago, Facultad de Medicina Clínica Alemana, Universidad del Desarrollo, Santiago 7610658, Chile; thomas.weitzel@gmail.com; 8 Instituto de Ciencias e Innovación en Medicina (ICIM), Universidad del Desarrollo, Santiago 7610658, Chile; 9 Centre for Biotechnology and Bioengineering, Universidad de Chile, Santiago 8370456, Chile; aolivera@ing.uchile.cl; 10 Facultad de Ciencias, Físicas y Matemáticas, Universidad de Chile, Santiago 8370456, Chile; 11 Biomolecular Simulations Group, Institut Pasteur de Montevideo, Montevideo 11400, Uruguay

**Keywords:** SARS-CoV2, variant, Covid19

## Abstract

The emergence of SARS-CoV-2 variants, as observed with the D614G spike protein mutant and, more recently, with B.1.1.7 (501Y.V1), B.1.351 (501Y.V2) and B.1.1.28.1 (P.1) lineages, represent a continuous threat and might lead to strains of higher infectivity and/or virulence. We report on the occurrence of a SARS-CoV-2 haplotype with nine mutations including D614G/T307I double-mutation of the spike. This variant expanded and completely replaced previous lineages within a short period in the subantarctic Magallanes Region, southern Chile. The rapid lineage shift was accompanied by a significant increase of cases, resulting in one of the highest incidence rates worldwide. Comparative coarse-grained molecular dynamic simulations indicated that T307I and D614G belong to a previously unrecognized dynamic domain, interfering with the mobility of the receptor binding domain of the spike. The T307I mutation showed a synergistic effect with the D614G. Continuous surveillance of new mutations and molecular analyses of such variations are important tools to understand the molecular mechanisms defining infectivity and virulence of current and future SARS-CoV-2 strains.

## 1. Introduction

In the past two decades, β-coronaviruses have caused three large-scale outbreaks. Severe acute respiratory syndrome coronavirus 2 (SARS-CoV-2) emerged in 2019 as the etiological agent of the severe respiratory coronavirus disease 2019 (COVID-19) [1,2] COVID-19 rapidly became a global pandemic with devastating effects. SARS-CoV-2 has taken millions of lives, as new cases steadily increase worldwide [3]. The first genome sequence was available in the early phase of the pandemic, since then, emerging variants with potential impact on viral fitness have been of scientific and public health concern [4,5,6,7].

In general, coronaviruses have a low sequence diversity due to their unique capacity to proofread and remove mismatched nucleotides during genome replication and transcription [8]. Nevertheless, the sequential accumulation of mutations affecting the spike protein has been reported in previous SARS outbreaks [9]. These variants may increase the protein’s affinity to its receptor (ACE2) and potentially impact transmission rates and disease severity [9,10,11]. During the SARS-CoV-2 pandemic, the dynamic tracking of variant frequencies revealed the emergence of a mutation (D614G), resulting in a worldwide shift of the structure of the spike protein [12]. The G614 variant rapidly superseded the D614 haplotype even in localities where the wildtype was established and showing a selective advantage [13]. Up to now, the analysis of spike sequences has revealed 400 distinct mutation sites. Although the functional impact of most of these mutations remain largely unknown, current evidence indicates that accumulation of certain mutations in the S protein might lead to higher affinity with host receptors and resistance against antibodies [6,11,12,14,15]. Therefore, tracking such changes in the structure of SARS-CoV-2 proteins through genomic surveillance becomes increasingly relevant as the pandemic evolves [12,16], in particular as the development of targeted therapies and vaccines may increase selection pressure on the virus evolution [17].

Geographically isolated populations can provide a natural laboratory environment for evolutionary studies regarding genomic changes and their epidemiological relevance [18]. Here, we describe the dynamics of SARS-CoV-2 mutations including the emergence of a novel haplotype in the Magallanes Region in sub-Antarctic Chile. A comparative set of coarse-grained molecular dynamics simulations of WT, D614G, and T307I/D614G mutants pinpointed coordinated motions between the N-terminal and the Receptor Binding Domains of Spike protomers and identified a previously unrecognized dynamic domain containing the mutation positions 307 and 614, along with the furin cleavage site.

## 2. Materials and Methods

### 2.1. Study Sites and Setting

This study was performed in the region of Magallanes and included cases from all the below mentioned localities. During the pandemic, the only available entry point was the main city of Punta Arenas (124,169 inhabitants), reachable by plane from the capital city Santiago, located at 2,193,000 km distance. The city of Puerto Natales (18,505 inhabitants) was reachable by a 246 km road trip from Punta Arenas. Porvenir (5907 inhabitants) is located on the Tierra del Fuego Island at 50 km distance, reachable by plane or ferry. Puerto Williams (1868 inhabitants) was connected by a 490 km flight from Punta Arenas.

### 2.2. Samples, Extraction of Genetic Material and Virus Detection

SARS-CoV-2 sequences were obtained from positive COVID-19 routine samples, diagnosed at the Laboratory of Molecular Medicine, Universidad de Magallanes in Punta Arenas, Chile. Nasopharyngeal swab samples were collected using the Genosur™ device according to manufacturing instructions. The extraction of viral genetic material was performed using Mag-Bind® Viral DNA/RNA 96 Kit (Omega, Cat. No M6246-03) in automated equipment MagEx Starlet (Hamilton). The viral RNA was reverse-transcribed and amplified for the ORF1, N and S genes by quantitative PCR with TaqMan™ 2019 nCoV Assay Kit v1 kit (Applied biosystems, Cat. No A47532, Foster City, CA, USA) in a AB7500 Fast thermocycler (Applied biosystems, Foster City, CA, USA) or in a LightCycler 480 II qPCR device (Roche). Additional sequences of 18 samples from the same region were obtained through GISAID, provided by the national reference center (Instituto de Salud Pública) [19].

### 2.3. Sequencing of SARS-CoV-2 Isolates from Clinical Samples

Amplification of the virus genome and the construction of libraries was performed using the protocol published by the ARTIC network [20], which allows sequencing by Oxford Nanopore Technologies (ONT) devices. Reverse transcription of viral RNA was performed using Random Hexamers (NEB, Cat. No S1230S, Ipswich, MA, USA) and SuperScript™ IV Reverse Transcriptase (ThermoFisher, Cat. No 18090010, Waltham, MA, USA). The cDNA generated was amplified by multiplex PCR using 218 primers distributed in two pools with Q5® Hot Start High Fidelity DNA polymerase (NEB, Cat. No M0493S, Ipswich, MA, USA) under the following cycling and temperature conditions: 1 cycle of 98 °C × 30 s; 35 cycles of 98 °C × 15 s and 65 °C × 5 min. The PCR products were ligated to native barcodes (EXP-NBD104, EXP-NBD114) and to nanopore adapters using the SQK-SLK109 sequencing ligation kit. The experiments were run on FLO-MIN106D flow cells. Sequencing experiments were performed in cells with >1000 active pores and the reading was carried out for 24 h. Orthogonal validation of detected variants and genotyping at T22482 position were performed from representative samples of 31 epidemiological weeks (March to November 2020). S gene PCR products were generated using primers 74 R and 74 L from version 3 of ARTIC protocol [20] and sequenced by capillary electrophoresis in a SeqStudio™ Genetic Analyzer (Applied Biosystems, Foster City, CA, USA, Thermo Fisher scientific, Waltham, MA, USA).

### 2.4. Genome Assembly and Phylogenetic Analysis

Genome assembly was performed following the ARTIC pipeline “nCoV-2019 novel coronavirus bioinformatics protocol v1.1.0”. For high-accuracy base calling and demultiplexing, Guppy v4.0.14+8d3226e was used. Reads quality was assessed with the pipeline mentioned above. The multi-tool platform Nextstrain [21] was used for genome alignment (MAFFT v7.4 [22]), phylogenetic analysis (IQ-TREE v1.6 [23]), and maximum-likelihood phylodynamic analysis (Treetime [24]).

### 2.5. Identification of Variants and Sequence Analysis

The ARTIC pipeline was also used to perform variant calling verifying the minimum quality of the identified variants. CoV-Seq was used for the annotation of the identified variants [25]. The assessment of substitution type (missense, silent or nonsense) was performed with Python scripts. Finally, in order to assess global prevalence of mutations identified in Magallanes, genomes and metadata were downloaded through GISAID and updated on 12 December 2020 [26]. The occurrence of these mutations was analyzed with scripts developed in Python.

### 2.6. Protein Modeling and Molecular Dynamics Analysis

Coarse-grained molecular dynamics simulations were performed for 10 µs using the SIRAH force field following the protocol reported by Machado et al. [27] Simulations and analyses were performed using GROMACS 18.2 [28]. The coordinates corresponding to the Protein Data Bank structure 6XR8 [17] were used and missing loops and mutations were added using the Swiss Model server (https://swissmodel.expasy.org, accessed on 7 October 2020) [29]. Glycans were removed from the structure. The coordinates were mapped from all-atoms to coarse-grain using the CGconv script provided with the SIRAH distribution. All proteins were placed in a simulation box, extending 2 nm in all the directions beyond each protein´s coordinate position. Proteins were solvated with coarse-grained water [30], and the ionic strength was set to 150 mM according to the methods proposed by Machado & Pantano [31]. The proteins were progressively relaxed as earlier described [27]. Non-bonded interactions were treated with a 1.2 nm cutoff and PME for long-range electrostatics. A time step of 20 fs was used. Snapshots were recorded every 200 ps for analysis. Principal component analysis was performed on the Calpha carbons of residues 25 to 237, 262 to 437, 507 to 614, 641 to 676, and 690 to 698 using GROMACS 2018.3 utilities. The backmapping of the coarse-grained coordinates to all-atoms was done with SIRAH Tools [27]. Multiple sequence alignment was performed with ClustalW v2.1, and visualized with MEGA v10.1.8. NCBI Reference sequences of spike proteins included in the analysis were YP_009724390.1 (SARS-CoV-2), YP_009825051.1 (SARS-CoV), YP_007188579.1 (MERS-CoV), AXT92528.1 (HCoV-HKU1), YP_009555241.1 (HCoV-OC43), QHR63300.2 (CoV-RaTG13), AGT17716.1 (M-CoV), YP_009113025.1 (HKU24). The structural alignments were performed using Swiss-PdbViewer [32], displayed by PyMOL v.2.4.1 [33], and rendered by Blender v.2.92.0 [34]. The trajectories generated in this study are available in the Zenodo database [35], as a part of the SIRAH-CoV2 initiative for data sharing [36].

### 2.7. Statistical Analyses

Linear regression was carried out to assess the association between the emerging variant and case incidence, intensive care unit admission, and mortality rates using R software version 4.0.

## 3. Results

### 3.1. Introduction and Spread of SARS-CoV-2 in the Magallanes Region

**Figure 1:**
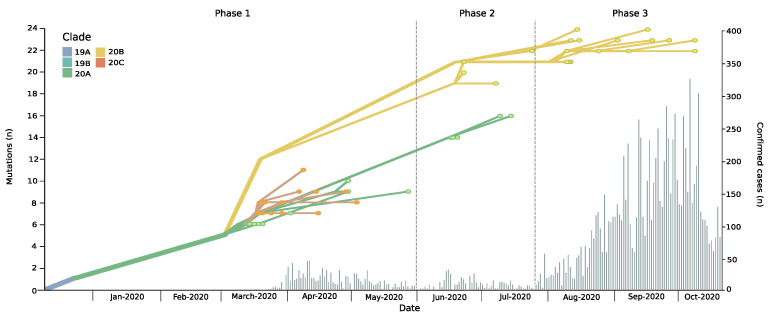
Phylodynamic evolution and new daily cases incidence in the Magallanes Region. The phylodynamic tree depicts the number of mutations of isolated genomes during the study period in the Magellanes Region; colors refer to Nextstrain clade classification (upper left corner) of analyzed strains. Clade defining mutations: 19A Wuhan Strain; 19B: ORF8 251S; 20A: Spike 614G; 20B: derived from 20A bearing Nuclocapsid 203K, N204R and ORF14 50N; 20C: derived from 20A bearing ORF3a 57H and ORF1a 265I. Histogram chart showing daily incidence rates of confirmed COVID-19 cases in the Magallanes Region.

This study was carried out in the world´s southernmost region between latitudes 45 and 55°S [18]. The geographically isolated situation of Magallanes, which is only accessible by ship, plane or through Argentina, facilitates the control of the circulation of inhabitants and visitors. Covid-19 was firstly introduced into the region on 17 March 2020, by a traveler from Santiago, followed by a substantial increase during following weeks and months (Figure 1). As a consequence, a series of restrictions were introduced from April 7 onwards, including an entry ban for non-residents and a mandatory 14-day monitored self-isolation for arrivals, as well as various physical distancing and lockdown measures. Restrictions were partially relaxed during May, but due to a subsequent progressive case increase, they were reintroduced in August. Strict restrictions and sanitary controls at the ports of entry and international borders were in place throughout the entire pandemic.

The epidemic in Magallanes can be divided into three phases. The first epidemic phase, which took place from mid-March to the end of May, was followed by a second wave during June and July (Figure 1). Respective average daily cases during these two periods were 14.1 and 9, with maximum daily cases reaching 46 and 32, respectively. Starting in end-July, Magallanes was hit by a third epidemic wave, with significantly higher daily case numbers (maximum, 324; average, 114). As of 30 December 2020, the Magallanes Region reported a total of 16,725 laboratory-confirmed SARS-CoV-2 cases, and with a total incidence rate of 10,043/100,000 inhabitants, it is among the most affected regions worldwide [3]. This had a dramatic impact on the healthcare situation, temporarily surpassing the ICU beds capacity and leading to aeromedical patient transfers to hospitals in less affected regions. Despite the increase in newly diagnosed cases there was not a significant change in the reproductive number that remained at an average of 1.08 (0.91–1.25).

### 3.2. Dynamic Tracking of Variants Through Infection Spreading Events

**Figure 2:**
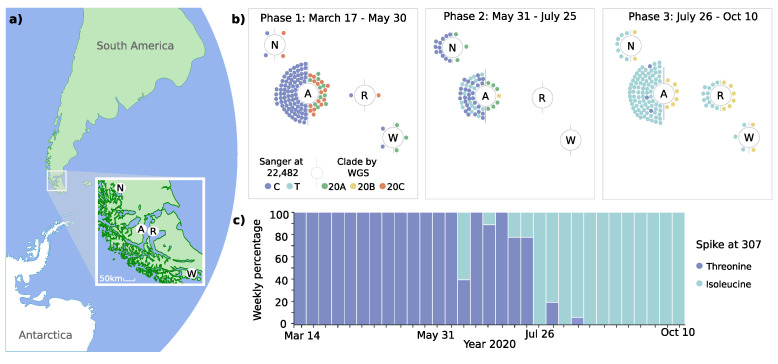
Geodynamic evolution of clades and spike 307 mutation. (**a**) Shows the geolocation of study sites in the Magallanes Region in Chile’s extreme south (N, Puerto Natales; A, Punta Arenas; R, Porvenir; W, Puerto Williams). (**b**) Demonstrates the distribution of mutations of spike protein at genomic position 22,482 and clades (Nextstrain classification) at the four study sites. Colored dots represent individual cases analyzed by targeted sequencing and whole genome sequencing (WGS). (**c**) Shows the relative frequency of spike protein mutations at position 307.

Genomic surveillance was performed in different localities of the Magallanes sub-Antarctic region, including Punta Arenas and Puerto Natales, the two main cities of the continental part, Porvenir on Tierra del Fuego, and Puerto Williams on the Navarino Island (Figure 2a).

We performed multiplex tiling PCR on 48 samples, followed by next-generation sequencing of indexed samples on MinIOn devices. On average, 349 ± 32 thousand reads were generated per sample, and virus genomes were assembled by mapping against the reference genome NC_045512 [37]. The assembly yielded 39 genomes with coverage higher than 95% (mean 99.07, range 95.34–100%). Together with further 18 sequences retrieved from GISAID [26] we analyzed 57 whole genomes.

The phylodynamic analyses revealed the predominance of distinct clades over time (Figure 1 and Figure 2b). During phase 1, three Nextstrain clade 20A variants (C3037T, C14408T, and A23403G) were identified, which accounted for 43.3% of cases. However, two variants of Nextstrain clade 20C (C1059T and G25563T) emerged during this initial phase (56.7%). During phase 2, variants of clade 20B (G28881A, G28882A, and G28883C) emerged, accounting for 45.5% of cases in this phase and 100% in phase 3. These changes occurred simultaneously in all four localities, over a maximum linear distance of 490 km (Figure 2a,b).

### 3.3. Emergence of a Novel Haplotype with Nucleocapsid and Spike Protein Mutations

**Figure 3:**
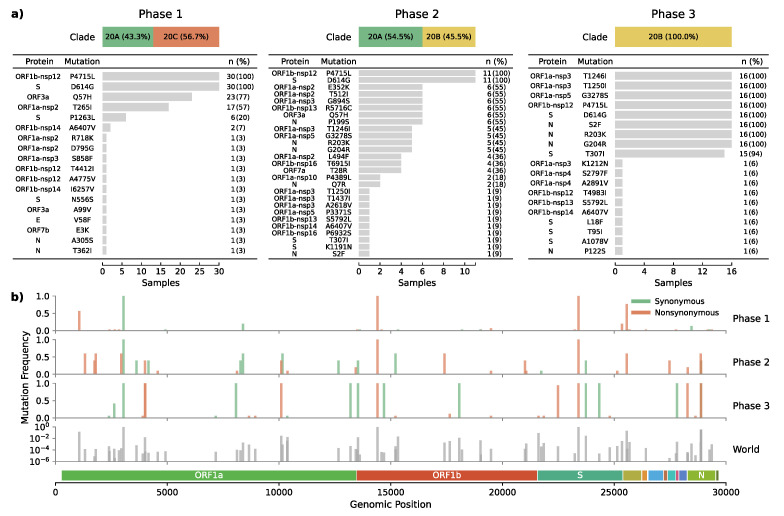
Mutational effect and frequency across epidemic phases. (**a**) Shows the frequency of amino acid changes induced by non-synonymous mutations. (**b**) Depicts the genomic position and relative frequency of both synonymous and non-synonymous mutations during the three epidemic phases the world’s global relative frequency included as a reference.

The emerging clade 20B haplotype was defined by nine co-occurring non-synonymous mutations present in 94% of the samples (*n* = 15/16) from the last phase. Four mutations were located within nonstructural proteins (Nsp3: T1246I, Nsp3: T1250I, Nsp5: G3278S, Nsp12: P4715L), three in the nucleocapsid (S2F, R203K, G204R), and two affecting the spike protein (D614G, T307I) (Figure 3). This novel haplotype, not reported from anywhere before, has since end-July replaced all other lineages in the Magallanes Region.

One of the haplotype-defining mutations was the spike T307I amino acid change caused by a C-to-T transition at position 22,482 (Figure 3b). This mutation had an early occurrence in April in Santiago de Chile (*n* = 1) and is present in other parts of the world such as USA (*n* = 56), UK (*n* = 35), China (*n* = 10), Australia (*n* = 2), Denmark (*n* = 2), India (*n* = 2), Japan (*n* = 2), Belgium (*n* = 1), Canada (*n* = 1), Netherlands (*n* = 1), and Norway (*n* = 1), resulting in an overall prevalence of only 0.004% (Figure 3b).

To increase the temporal resolution of the analysis of the spike protein coding region, we amplified and sequenced a total of 212 samples at position T22,482. The T307I mutation was firstly detected on June 11 and subsequently co-existed with the unmutated T307 variant (Figure 2c). While the mutation was not present in phase 1, it was observed in 31.6% of cases in phase 2 and predominated with 97.2% in phase 3.

A significant correlation was found between the proportion of samples with the T22482 mutation and the weekly incidence rate of new cases (β = 758.082, *p* = 0.00022), the association was attenuated when adjusting the model by the positivity testing rate (β = 313.46, *p* = 0.00557). There was no association with the mortality rate and there was an inverse correlation with the severity of the cases as assessed by the proportion of ICU bed occupation over active cases (r(28) = −0.62, *p* = 0.00025).

### 3.4. Structural and Molecular Dynamic Analysis of T307I

The worldwide lineage shift to the 614G variant has drawn attention to spike protein mutations [12]. Therefore, we further analyzed possible structural changes of this protein in the here described haplotype emerging in Magallanes. The spike protein is a homotrimer decorating the outer surface of the virus, where each chain is anchored to the viral membrane by a single transmembrane motif. Each protomer is composed by subunits S1 and S2 [38]. The specific recognition of the S1 receptor binding domain (RBD) with the N-terminal lobe of isoform 2 of human angiotensin converting enzyme (ACE2) triggers a conformational change that mediates the shedding of S1 and insertion of S2 fusion peptide into the host membrane. This process is accompanied by the enzymatic cleavage of S1/S2 polypeptide chains by a host´s furin [39].

**Figure 4:**
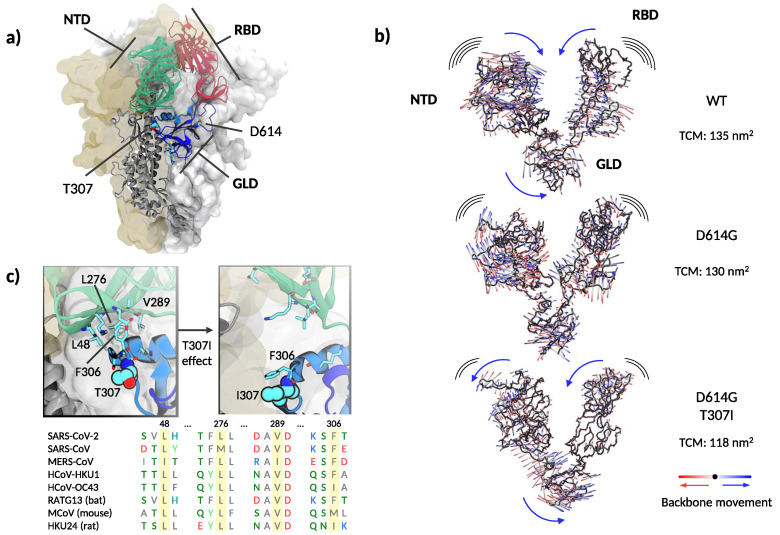
Structure and dynamics of the soluble part of SARS-CoV-2 spike. (**a**) Global architecture of the homotrimer. The foremost protomer is shown in cartoon representation, while the other two are shown by solvent accessible surface. The S1 segment is colored by structural domains discussed in the main text (green: N-terminal domain, NTD; red: receptor binding domain, RBD; blue: gear-like domain, GLD). The two amino acids mutated are shown in space-filling representation and the amino acids in the furin loop are shown as sticks. (**b**) Extreme projections of the main component of the motion in the S1 segment. The black tube corresponds to the average position of the protein backbone. The direction of the motion is indicated by lines changing from red to blue color. (**c**) Upper left panel: close up on the amino acids surrounding T307 and F306 in the cryo–electron microscopy structure used as the starting configuration. Upper right panel: Final conformation of the molecular dynamics simulation with the side chains of I307 and F306 in close contact. Lower panel: spike protein alignment around the conserved hydrophobic cluster (L48, L276, V289) interacting with F306 in different β-coronaviruses.

Threonine 307 is part of the S1 segment located downstream to the N-terminal domain (NTD, Figure 4a), this segment is regarded as the linking region between NTD and RBD [40]. The polypeptide between amino acids 306–331 and 528–686 forms a folding domain with the C-terminal part of S1. Remarkably, the same folding domain contains T307, G614, and the key furin cleavage motif (Figure 4a). This domain is apposed to the S2 part of a neighboring protomer in the quaternary arrangement and likely modulates S1–S2 interactions [38]. Because of its dynamic behavior, as detailed below, we will refer to this linking domain as “gear-like” domain (GLD).

Recent evidence suggests that the D614G mutation alters the equilibrium between RBD-closed and RBD-open conformations of the spike protein [41]. However, the precise molecular mechanisms regulating RBD states to prime the interaction with ACE2 are subject to controversy [6,40,41,42]. Molecular dynamics simulations at different degrees of resolution are extensively applied as computational microscopes to unravel the structural and dynamical impact of mutations and posttranslational modifications on the spike protein [43,44,45]. Here, we performed coarse-grained molecular dynamics simulations of the spike trimer’s soluble domain for the WT, D614G, and the double mutant T307I-D614G. The three variants showed a similarly high dynamic behavior with root-mean-square deviations (RMSD) from the starting position of nearly 0.7 nm, accompanied by a rapid loss of the initial symmetry between protomers. However, we did not observe a clear opening of the RBDs in the 10 microseconds-long trajectories. The presence of several long and flexible loops along the polypeptide chains made it challenging to dissect clear conformational differences. This may agree with the reported structural similarity between WT and D614G [46]. However, principal components analysis on the structured regions of the S1 segment showed a concerted revolving motion, in which the NTD of one protomer moves against the RBD of the neighboring protomer (see Supplementary Videos S1 and S2). The folding domain harboring both mutations acts analogous to a central gear connecting the motion between the NTD and RBD. Qualitatively, the spike WT, the D614G, and the D614G-T307I variants showed the same behavior, although with different amplitudes. The trace of the covariance matrixes (which provide a quantitative gauge of the movement’s variance) calculated on the S1 segment resulted in 135 nm${}^{2}$, 130 nm${}^{2}$, and 118 nm${}^{2}$ for the WT and the D614G and T307I-D614G mutants, respectively. Indeed, a comparative view of the extreme projections of the movement in the three proteins showed a progressive decrease of mobility from WT to single and double mutation (Figure 4b, and Supplementary Video S3).

Analyzing further distinctive features among the threelmutants, we noticed that T307 is solvent-exposed and flanked by F306, which participates in a well-conserved hydrophobic cluster with L48, L276, and V289 within the NTD (Figure 4c).

**Figure 5:**
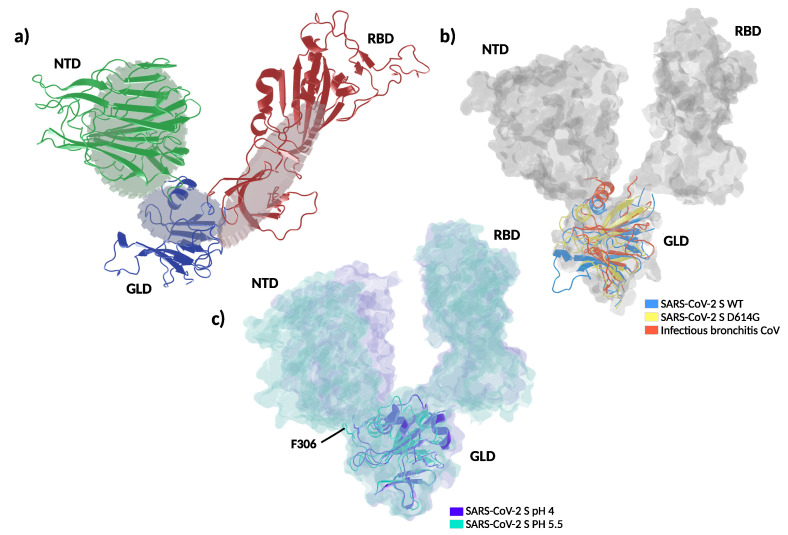
Proposed molecular dynamic of spike protein. (**a**) Schematic representation depicts a proposed gear-like mechanism for the molecular dynamic of the S1 subunit in which the rotation of gear-like domain (GLD) is accompanied by the solidary movement of the N-terminal and receptor binding domains (NTD and RBD respectively). (**b**) Superposition of spike experimental structures in the all-RBD-down state captured with different rotations of the GLD, depicting a counter-clockwise rotation of the gear-like domain. The GLD is shown in cartoon representation and NTD and RBD from the wild type protein are shown in solvent accessible surface. In blue: SARS-CoV-2 S wild type (WT) (pdb: 6XR8), yellow: SARS-CoV-2 S D614G (pdb: 6XS6), red: infectious bronchitis coronavirus (pdb: 6CV0). (**c**) Superposition of spike experimental structures in the all-RBD-down, depicting domain movements between pH 5.5 and 4.0. In purple: spike at pH 4.0 (pdb: 6XLU), calypso: spike at pH 5.5 (pdb: 6XM5). F306 are shown as sticks. NTD: N-terminal domain, RBD: receptor binding domain, GLD: gear-like domain.

Molecular dynamic simulations revealed a close attractive interaction between F306 and the hydrophobic cluster in the WT and D614G mutant (Figure 4c left image). This non-covalent bond was deranged in the double D614G-T307I mutant through the change from a polar to a hydrophobic amino acid at position 307, resulting in a permanent removal of F306 from its hydrophobic cluster and in a new attractive interaction with the mutated isoleucine in position 307 (Figure 4c right image and Supplementary Video S4). The detachment of F306 results in a reduced mobility of the S1 segment and a change in the rotation direction of NTD (Figure 4b,c, Supplementary Videos S3 and S4), suggesting a mechanism similar to a dislocated gear, thus interfering with the coupling of NTD and RBD (Figure 5b).

Coarse-grained molecular dynamics simulations and the absence of glycosylation in our setup might limit our conclusions. Therefore, we sought to look for support to the gear-like domain hypothesis in published experimental results. Amino acid sequence alignments showed that the hydrophobic cluster is highly conserved across different β-coronaviruses (Figure 4c). To analyze the structural effect of the genetic variations within the GLD, we compared the WT and D614G to the avian infectious bronchitis virus, which contains a glycine at the homologous position of F306 [47]. This analysis revealed that the genetic variety of the hydrophobic cluster results in different positioning of the alpha helix connecting GLD to NTD (Figure 5b). On the other hand, it has been recently reported that low pH values markedly favor all-RBD-down states [48]. A close inspection of the spike structures at different pH values reveals two compelling features. First, passing from pH = 4 to pH = 5.5 involves a counter-clockwise rotation of the GLD (Figure 5c). Second, the stretch of amino acids 825 to 858 loses mobility at pH = 4, acquiring a helical structure. Interestingly, this segment gets in close contact with the GLD in acidic conditions. In terms of our model, this stretch acts as a wedge in the gear, blocking the NTD and RBD conformations in a closed state.

## 4. Discussion

The present study analyzed the molecular evolution of SARS-CoV-2 haplotypes in the Magallanes Region in Chile´s extreme south. This remote area is characterized by strict controls at entry points facilitated by isolation imposed by its geoclimatic characteristics. Our molecular analyses revealed the initial introduction of clade 20 strains as observed in other regions in Chile [19]. However, over a period of six weeks, a novel SARS-CoV-2 haplotype carrying the T307I spike mutation replaced all competing lineages that were previously established. This phylodynamic shift was observed simultaneously in all four localities within the region. Noteworthy, this change was associated with a significant increase in infection rates resulting in one of the worlds’ highest incidences.

Spontaneous mutations can rise in frequency due to natural selection, random genetic drift, or epidemiological factors. Because these forces often coexist, it is challenging to unambiguously attribute an emerging viral mutation to certain advances in viral fitness or random selection processes [49]. The great majority of mutations that accumulate in viral pathogens during replication and viral spread are, in fact, of little functional relevance. However, genetic selection is observed during antigenic drift to escape preexisting immunity or during pathogen adaptation to a new host [4,50,51,52]. In the ongoing epidemic in Magallanes, we observed a complete lineage shift with a new haplotype that accounted for all analyzed cases during the last 3 months of the study period. This new haplotype coexisted with other variants for several weeks, replacing established variants in all four cities, and was associated with a dramatic increase of daily case numbers, despite the fact that control measures being already in place. Although geographical isolation may contribute to a lower diversity and founder effect cannot be excluded, this data may suggest that the novel variant acquired certain evolutionary benefits.

Recently, the United Kingdom has reported that a large proportion of new cases in South East England belonged to a new single phylogenetic cluster defined by multiple spike protein mutations (deletions 69–70 and 144, N501Y, A570D, D614G, P681H, T716I, S982A, D1118H) [53]. Similarly, as observed in our study, this lineage shift coincided with a rapid increase in COVID-19 cases. Although establishing causality remains challenging, these findings reinforces the need of active genomic surveillance across the globe [53].

The emerging variant contained nine missense substitutions within Nsp, nucleocapsid and spike proteins. Two threonine-isoleucine substitutions (T1246I, T1250I) were localized within Nsp3. This papain-like protease is required during the polyprotein cleavage and also has been associated with evasion mechanisms against antiviral host immune responses [54]. Another mutation was located within Nsp5, a protease that has been the subject of drug repurposing studies [55]. Three variants were located within the N-protein coding gene, two of these polymorphisms refer to amino acid changes (R203K and G204R), which have been associated earlier to increased fitness and adaptation [56]. Potential effects of the third mutation, inducing a S2F amino acid change, are as yet unknown.

Due to the putative role of spike mutations in virus spreading [6,12,17], we further investigated the effects of the T307I substitution on the spike protein structure. Amino acid 307 lies on the opposite side of D614G in the same folding domain being completely solvent-exposed. Our simulations showed that the polar-to-hydrophobic T307I replacement pulls the preceding F306 and removes it from a highly conserved hydrophobic cavity of the N-terminal domain. We therefore postulate that upon T307I substitution, F306 is attracted to the mutated I307 to optimize hydrophobic interactions. To substantiate this speculation, we performed coarse-grained molecular dynamics simulations of the spike trimer’s soluble part introducing the T307I and D614G mutations. As expected, we observed the detachment of F306 from its hydrophobic cluster to interact with I307. This event reduced the global movement of the S1, and may be reminiscent of a cog dislocation in a gear mechanism. According to this model, the gear-like domain (GLD)—where D614G and T307I mutations are located-would convey the motion between NTD and RBD (Figure 5a). Despite the coarseness of our approach and the absence of glycosylation in our simulations, the relevance of the F306’s hydrophobic cluster in modulating NTD-RBD reciprocal conformation was further highlighted by the high amino acid conservations across different β-coronaviruses as shown in Figure 4c. Further supporting our model, the superposition of spike structures obtained by electron microscopy suggested a different rotation on the positioning of the GLD in the D614G compared to the WT. A rotation in the opposite direction can be observed in infectious bronchitis virus [47], where the F306 is naturally replaced by a glycine (Figure 5b). Similarly, the determination of the spike structures at different pH values captured the GLD in different rotations, with the segment between amino acids 825 to 858 adopting a well defined structure that acts as a wedge in the gear, blocking the spike in the all-RBD-down conformation.

Our findings contribute to the mechanistic view of Yurkovetskiy et al., who suggested that the D614G mutation unlocks a latch between this domain and the RBD [41]. Furthermore, the presence of the two mutations in the same folding domain containing the furin cleavage loop could affect the dynamics of the S1/S2 dissociation.

Whereas mutations emerging within the receptor binding domain such as N501Y and N440K might have a direct impact on ACE2 binding [11], our work further supports the idea that distant variations affecting RBD dynamics, such as D614G, must also be monitored. Indeed, the fact that the spike conformation is highly susceptible to mutations at sites of contact between the S1 and S2 subunits, has been proposed as one of the molecular basis of increased infectivity in SARS-CoV-2 mutants [57]. Our findings are consistent with recent experimental evidence describing regions of S1 acting as a hinge for RBD up-movement [58,59]. Moreover structure analysis of mutations within the GLD demonstrate that the disruption of the interaction between S1 and S2 subdomains result in different ratios of “up” and “down” states of the RBD [59]. To interact with the ACE2 receptor the RBD needs to adopt “up” conformations, which are primarily achieved through RBD movement combined with smaller alterations in neighboring domains. RBD positioning seems to be mediated by pH through coordinated movements of three key spike regions [48]. Two of these regions are located within the GLD and one within the S2 segment. Our analysis suggest that the conformational changes induced by the T30I mutation are analogous to those induced by pH as assessed by cryo-electron microscopy analysis. In addition, these mechanistic changes are proposed as molecular basis for immune evasion [48].

The mechanisms by which the above-described variations of the spike protein would affect virulence and infectivity remain unclear. The T307I mutation may promote RBD-open conformations, similarly as proposed for D614G [14], or decouple the mechanic communication between N-terminal and RBD. Either effect may result in an increased ratio of prefusion vs. postfusion spike proteins [17]. Virions with a higher proportion of prefusion proteins are supposed to have increased infectivity [17]. This might explain the increased incidence observed after the epidemiological switch in our study. In addition, Cai et al. have suggested an immune evasion role of postfusion spike trimers [17]. According to this hypothesis, a haplotype exposing less spike trimers in postfusion state would result in a more efficient host response, which is in accordance with the lower rate of severe infections observed in our study.

At the time of writing, the reported haplotype seems to remain locally contained, which could be attributed to the geographical isolation and/or higher fitness of other mutants. Interestingly, variants of interest and variants of concern currently emerging in different parts of the world show non-synonymous mutations within the here described GLD. The B1.525 variant harbors a Q677H mutation [60], the variant B1.1.7 reported in the United Kingdom carries A570D and P681H mutations [61], the P1 variant from Brazil shows the H655Y mutation [62], and the recently reported B.1.617 strain in India shows a P681R change [63]. Whether these mutations located within the same domain as T307I may induce a similar impact on the molecular dynamics warrants further investigation. Other mutations located around the gear domain have also been proposed as drivers of ongoing viral adaptation (Supplementary Figure S1) [64]. Mutations A653V and I692V, located inside the hydrophobic core of the gear domain, probably cause loss of compactness of the beta-sandwich core. Mutation H655Y alters the hydrophobicity of an exposed residue, with a less evident effect. Mutation T307I is unique in its location and interaction with the alpha-helix of the gear domain. Although this points out to a rather different effect of T307I in comparison with other reported mutations in the gear domain, it is possible that the loss of core compactness caused by A653V and I692V, and the change in hydrophobicity induced by H655Y could have similar dynamic effects as T307I.

Our analysis of genetic variants together with epidemiological data in a confined geographical region contributes to the growing knowledge of emerging SARS-CoV2 variants. The rapid shift to a mutated strain with a novel spike protein variation (D614G/T307I) suggested an adaptive advantage. Our analyses describe how molecular dynamic effects induced by mutations may affect the critical linking domain of the spike protein. According to this model, the observed double mutation leads to a reduced mobility of the S1 subunit, presumably increasing binding capacity and infectivity, which would explain the observed evolutionary advantage.

## Data Availability

Genomic data of newly sequenced samples are deposited in the GISAID with accession numbers provided in Supplementary Table S1.

## Supplementary Materials

The following are available online at https://www.mdpi.com/article/10.3390/v13050883/s1. Table S1: De novo assembled genomes including sample date, location and corresponding GSAID submission numbers, Figure S1: Comprehensive view of the location of recently reported spike mutations, Videos S1 and S2: Upper and side views of the motion between initial and final structures of the double mutated spike protein, Video S3: Projection of the principal component of the motion of the segment S1 for the three spike variants, Video S4: Morphing between initial and final conformations of the spike protein mutated on T307I-D614G.

## Abbreviations

The following abbreviations are used in this manuscript:

RBD	Receptor binding domain
NTD	N-terminal domain
GLD	Gear-like domain
