# Supplementary material for: Mutation in a SARS-CoV-2 Haplotype from Sub-Antarctic Chile Reveals New Insights into the Spike’s Dynamics
Source: Viruses. 2021 May 11;13(5):883. doi: 10.3390/v13050883 (PMC8151058; doi:10.3390/v13050883)
Supplement: Supplementary file 1 [file viruses-13-00883-s001.zip › Supplementary Materials for J.Gonzalez SARS-CoV2-V3.3.pdf]

Supplementary Materials for

Mutation in a SARS-CoV-2 Haplotype from sub-Antarctic Chile Reveals New Insights into the Spike's Dynamics

Jorge González-Puelma, Jacqueline Aldridge, Marco Montes de Oca, Mónica Pinto, Roberto Uribe-Paredes, Jose Fernández Goycoolea, Diego Alvarez-Saravia, Hermy Alvarez, Gonzalo Encina, Thomas Weitzel, Álvaro Olivera Nappa, Rodrigo Muñoz, Sergio Pantano, Marcelo A. Navarrete

Supplementary Table S1

Table containing *de novo* assembled genomes including sample date, location and corresponding GSAID submission numbers.

sample_id	***gisaid_id***	***sample_date***	***sample_location***
Chile/MA-ISPCH-1/2020	EPI_ISL_445268	2020-03-14	Punta Arenas
Chile/MA-ISPCH-2/2020	EPI_ISL_445269	2020-03-15	Punta Arenas
Chile/MA-ISPCH-3/2020	EPI_ISL_445280	2020-03-17	Punta Arenas
Chile/MA-ISPCH-6/2020	EPI_ISL_445284	2020-03-19	Punta Arenas
Chile/MA-ISPCH-4/2020	EPI_ISL_445282	2020-03-19	Punta Arenas
Chile/MA-ISPCH-5/2020	EPI_ISL_445283	2020-03-19	Punta Arenas
Chile/MA-ISPCH-16/2020	EPI_ISL_445301	2020-03-20	Punta Arenas
Chile/MA-ISPCH-12/2020	EPI_ISL_445293	2020-03-20	Punta Arenas
Chile/MA-ISPCH-8/2020	EPI_ISL_445289	2020-03-20	Punta Arenas
Chile/MA-ISPCH-7/2020	EPI_ISL_445288	2020-03-20	Punta Arenas
Chile/MA-ISPCH-10/2020	EPI_ISL_445291	2020-03-21	Punta Arenas
Chile/MA-ISPCH-13/2020	EPI_ISL_445294	2020-03-21	Punta Arenas
Chile/MA-ISPCH-14/2020	EPI_ISL_445295	2020-03-21	Punta Arenas
Chile/MA-ISPCH-9/2020	EPI_ISL_445290	2020-03-21	Punta Arenas
Chile/MA-ISPCH-15/2020	EPI_ISL_445299	2020-03-22	Punta Arenas
Chile/MA-ISPCH-11/2020	EPI_ISL_445292	2020-03-22	Punta Arenas
Chile/MA-CMM-31600/2020	EPI_ISL_459861	2020-03-25	Puerto Natales
Chile/MA-CADIUMAG-1/2020	EPI_ISL_681673	2020-03-30	Punta Arenas
Chile/MA-ISPCH-17/2020	EPI_ISL_445372	2020-03-30	Punta Arenas
Chile/MA-CADIUMAG-8/2020	EPI_ISL_681674	2020-04-03	Puerto Williams
Chile/MA-CADIUMAG-9/2020	EPI_ISL_681675	2020-04-07	Punta Arenas
Chile/MA-CADIUMAG-10/2020	EPI_ISL_681676	2020-04-09	Punta Arenas
Chile/MA-CADIUMAG-11/2020	EPI_ISL_681677	2020-04-15	Punta Arenas

Chile/MA-CADIUMAG-12/2020	EPI_ISL_681678	2020-04-16	Porvenir
Chile/MA-CADIUMAG-13/2020	EPI_ISL_681679	2020-04-29	Punta Arenas
Chile/MA-CADIUMAG-14/2020	EPI_ISL_681680	2020-04-30	Puerto Williams
Chile/MA-CADIUMAG-15/2020	EPI_ISL_681681	2020-04-30	Puerto Williams
Chile/MA-CADIUMAG-16/2020	EPI_ISL_681682	2020-04-30	Punta Arenas
Chile/MA-CADIUMAG-17/2020	EPI_ISL_681683	2020-05-04	Punta Arenas
Chile/MA-CADIUMAG-38/2020	EPI_ISL_681684	2020-05-28	Punta Arenas
Chile/MA-CADIUMAG-37/2020	EPI_ISL_681685	2020-06-17	Punta Arenas
Chile/MA-CADIUMAG-18/2020	EPI_ISL_681686	2020-06-18	Punta Arenas
Chile/MA-CADIUMAG-23/2020	EPI_ISL_681688	2020-06-20	Punta Arenas
Chile/MA-CADIUMAG-22/2020	EPI_ISL_681687	2020-06-20	Punta Arenas
Chile/MA-CADIUMAG-25/2020	EPI_ISL_681691	2020-06-23	Puerto Natales
Chile/MA-CADIUMAG-24/2020	EPI_ISL_681690	2020-06-23	Puerto Natales
Chile/MA-CADIUMAG-26/2020	EPI_ISL_681689	2020-06-23	Puerto Natales
Chile/MA-CADIUMAG-27/2020	EPI_ISL_681692	2020-07-08	Puerto Natales
Chile/MA-CADIUMAG-28/2020	EPI_ISL_681693	2020-07-10	Punta Arenas
Chile/MA-CADIUMAG-29/2020	EPI_ISL_681694	2020-07-15	Punta Arenas
Chile/MA-CADIUMAG-30/2020	EPI_ISL_681695	2020-07-25	Punta Arenas
Chile/MA-UMAG-Catg-2/2020	EPI_ISL_625681	2020-08-10	Porvenir
Chile/MA-UMAG-Catg-3/2020	EPI_ISL_625682	2020-08-10	Porvenir
Chile/MA-CADIUMAG-31/2020	EPI_ISL_681696	2020-08-11	Porvenir
Chile/MA-CADIUMAG-32/2020	EPI_ISL_681697	2020-08-12	Punta Arenas
Chile/MA-CADIUMAG-33/2020	EPI_ISL_681698	2020-08-12	Porvenir
Chile/MA-CADIUMAG-34/2020	EPI_ISL_681699	2020-08-12	Porvenir
Chile/MA-CADIUMAG-35/2020	EPI_ISL_681700	2020-08-15	Punta Arenas
Chile/MA-CADIUMAG-36/2020	EPI_ISL_681701	2020-08-16	Punta Arenas
Chile/MA-UMAG-Catg-19/2020	EPI_ISL_625680	2020-08-25	Punta Arenas

Chile/MA-UMAG-Catg-7/2020	EPI_ISL_625675	2020-09-03	Porvenir
Chile/MA-UMAG-Catg-20/2020	EPI_ISL_625676	2020-09-08	Punta Arenas
Chile/MA-UMAG-Catg-5/2020	EPI_ISL_625673	2020-09-17	Puerto Natales
Chile/MA-UMAG-Catg-21/2020	EPI_ISL_625677	2020-09-19	Punta Arenas
Chile/MA-UMAG-Catg-6/2020	EPI_ISL_625674	2020-09-27	Puerto Natales
Chile/MA-UMAG-Catg-4/2020	EPI_ISL_625678	2020-10-09	Puerto Williams
Chile/MA-UMAG-Catg-5-2/2020	EPI_ISL_625679	2020-10-09	Puerto Williams

Supplementary Figure S1

(a)

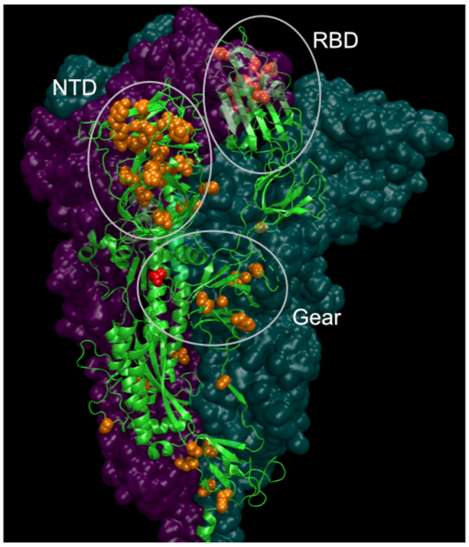

(b)

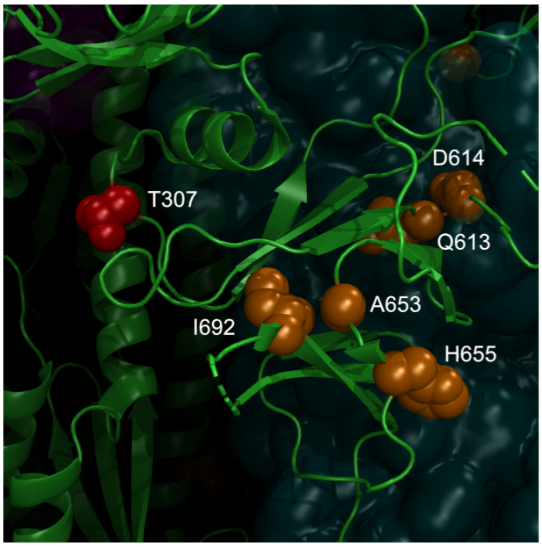

Supplementary Figure S1: Amino acid substitutions seen in currently circulating variants of concern and variant of concern-like viruses, as reported by Peacock et al. [64] and T307I (red) mapped onto PDB structure 6XR8. (a) Substitutions (orange) mapped onto one spike protomer (light green), showing the position of the RBD, NTD and gear domains. (b) Close-up of the gear domain showing positions T307, Q613, D614, A653, H655 and I692 (orange), in relation to position T307 (red).

Supplementary videos:

Supplementary Video S1: Upper view of the motion of the S1 segment. The X-ray structure of each protomer is shown in static cartoon representation. The animation shows a morphing (linear interpolation of the 3D positions) between the initial and final coordinates of the WT simulation. The morphing is shown only on the S1 domain of chain A for visual clarity. The T307 and D614 positions are shown in space filling representation for reference. N-terminal domain in light blue, receptor binding domain in red, and “gear-like” domain in blue.

Supplementary Video S2: Frontal view of the motion of the S1 segment. The X-ray structure of each protomer is shown in static cartoon representation. The animation shows a morphing (linear interpolation of the 3D positions) between the initial and final coordinates of the WT simulation. The morphing is shown only on the S1 domain of chain A for visual clarity. The T307 and D614 positions are shown in space filling representation for reference. N-terminal domain in light blue, receptor binding domain in red, and “gear-like” domain in blue.

Supplementary Video S3: Projection of the extreme motions on the S1 segment of each simulation. The WT, D614G, and T307I/D614I variants are shown from top to bottom. Colors as in Figure 4b.

Supplementary Video S4: Morphing between initial (wild type) and final (T307I) structures showing the close neighborhood of F306 and I307. See text for details, colors as in Figure 4c.
