# Supplementary figures and images for: Mutation in a SARS-CoV-2 Haplotype from Sub-Antarctic Chile Reveals New Insights into the Spike’s Dynamics
Source: Viruses. 2021 May 11;13(5):883. doi: 10.3390/v13050883 (PMC8151058; doi:10.3390/v13050883)

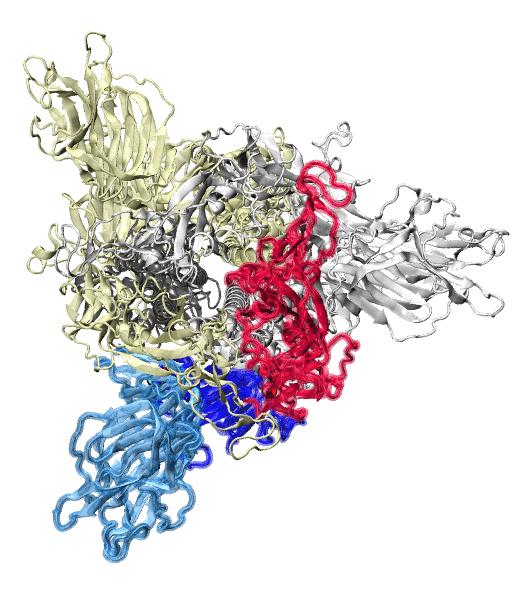

Supplement: Supplementary file 1 [file viruses-13-00883-s001.zip › SupplementaryMovie_1.gif]

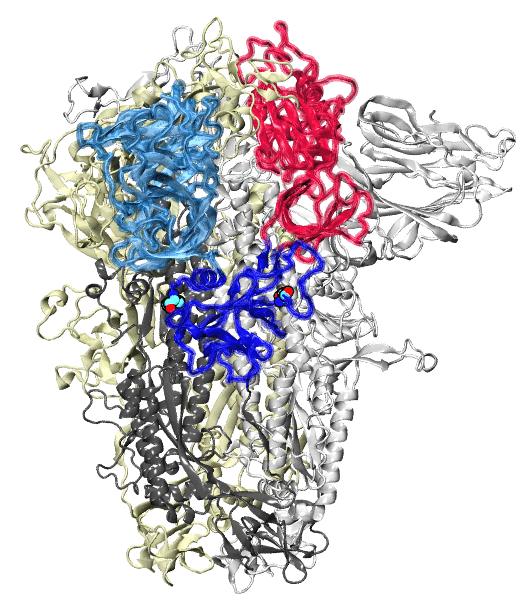

Supplement: Supplementary file 1 [file viruses-13-00883-s001.zip › SupplementaryMovie_2.gif]

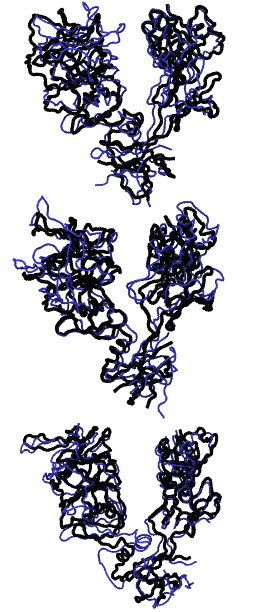

Supplement: Supplementary file 1 [file viruses-13-00883-s001.zip › SupplementaryMovie_3.gif]

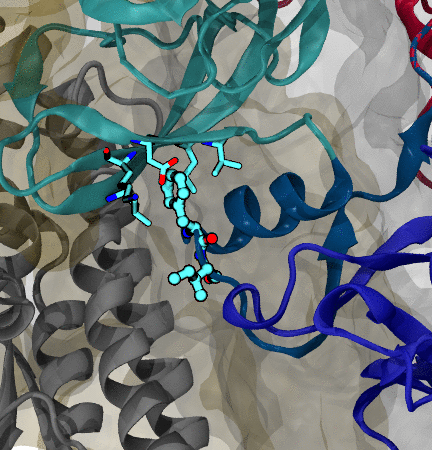

Supplement: Supplementary file 1 [file viruses-13-00883-s001.zip › SupplementaryMovie_4.gif]
